# An Analysis of the Health Effects of Physical Activity due to Active Travel Policies in Rennes, France

**DOI:** 10.12688/wellcomeopenres.20917.1

**Published:** 2024-03-19

**Authors:** Henry Fremont, Samuel Younkin, Anne Roué Le Gall, Natalie Levine, Jonathan Patz

**Affiliations:** 1 Nelson Institute for Environmental Studies, University of Wisconsin-Madison, Madison, Wisconsin, USA; 2 Département Santé-Environnementale, École des Hautes Études en Santé Publique, Rennes, France

**Keywords:** Health-Oriented Transportation, Active Travel, Active Transportation, Public Health, Physical Activity, Sustainability, Walking, Cycling

## Abstract

**Background:**

Rennes, a midsize city in France, features many opportunities for active travel. City officials seek to increase walking and cycling by 2030 to improve public health. Physical inactivity, a leading risk factor for premature mortality around the globe, has been shown to be associated with many chronic diseases including heart disease, type 2 diabetes, and cancer.

**Methods:**

Using the 2018 household travel survey of Rennes residents, we apply the Health-Oriented Transportation statistical model to assess health impacts associated with population-level rates of walking and cycling. We consider two proposed mobility and climate objectives which outline sustainable transportation goals by 2030. These include a shift in transportation mode share to increase walking and cycling trips, as well as a broad reduction in vehicle miles traveled (VMT) across the metropolitan area.

**Results:**

Our regression analysis demonstrated that factors of household car access and inner-city residency were predictors of prevalence (observed one-day proportion engaging in walking or cycling), participation (weekly proportion), and intensity (mean individual physical activity achieved through walking/cycling) of active travel. Age and education were additionally associated with prevalence. The 2030 mobility objective (mode share: 9% cycle, 35% walk) was associated with a reduction of 1,051 DALYs (disability-adjusted life-years), translating to $73 million USD ($23-$177) in averted costs. The climate objective (10% reduction in VMT) was associated with a reduction of 369 DALYs when replaced entirely by walking and 714 DALYs with cycling, translating to $26 million ($8-$62) and $50 million ($15-$121) saved, respectively.

**Conclusions:**

Rennes residents experience high participation in active travel, particularly those in the inner city. If residents achieve the city’s active travel goals for 2030, there is potential for a large reduction in health burden and subsequent costs. Reaching these goals may require significant investment in transportation programming and infrastructure to improve active travel opportunities.

## Introduction

Physical inactivity is the fourth leading risk factor for premature death across the globe ^1^. Insufficient physical activity is associated with higher all-cause mortality and incidence of non-communicable diseases, like cardiovascular disease and some cancers, as well as a multi-billion USD global economic burden ^1– 3^. Increased physical activity can not only attenuate these health risks, but lead to health co-benefits like improved sleep quality, prevention of injuries from falls, and cognitive improvements ^3, 4^. Environmental co-benefits of increased physical activity, particularly through increasing active travel methods, include large reductions in carbon emissions and improved air quality ^5^.

Active travel, or transportation by walking, cycling, or other active methods, can be an integral aspect of keeping a community active and healthy. Active travel is consistently associated with reductions in all-cause mortality, cardiovascular disease, and other non-communicable diseases, even after adjusting for demographic factors, recreational physical activity, and other comorbidities ^6^. Active travel is also associated with benefits to mental health, reduced stress, and improved social measures ^7, 8^. While benefits from active travel exist globally and across age groups, there is limited research on the different impacts across socio-economic groups and race/ethnicity populations ^9, 10^.

This article aims to consider active travel-related health outcomes in an urban environment using the Health-Oriented Transportation (HOT) statistical model ^11^. The HOT model considers scenarios in which an urban population achieves physical activity through transportation, i.e. *active travel*. The model is a transparent and easily accessible tool that allows users to assess the current and potential health benefits of active travel using data from a one-day travel survey ^11^. These open and communicable methods allow researchers and policymakers to be informed about potential health outcomes of various transportation policies, even when there is limited data availability. The HOT model was recently used to explore discrepancies in health outcomes based on different socioeconomic characteristics ^9^. For the current study, the HOT model is applied to data from the city of Rennes, France.

Rennes, a mid-size city in northwest France, features many opportunities for physical activity ^12^. Rennes policymakers have an opportunity to greatly improve health through investment in active transportation for the nearly 300,000 adult population, as measured in 2018 ^13^. In 2022, the city committed 7% of its entire budget to a wide range of actions to promote physical activity among its citizens, such as sports facilities and active mobility interventions ^14^. To aid attainment of the Rennes global transport policy that focuses on simultaneously reducing air pollution and promoting active mobility, the city commissioned the Household Travel Survey in 2018, which is used in the current analysis ^14^. Rennes was selected for these analyses both for the cities’ prioritization of transport and health as well as a recent research collaboration under the Complex Urban System for Sustainability and Health (CUSSH) project with the École des Hautes Études en Santé Publique (EHESP) ^15^.

In addition to our assessment of walking and cycling in Rennes using the Health-Oriented Transportation statistical model, we employ novel methods to compute health impacts associated with two proposed policies affecting transportation mode share in an urban environment ^16^. We consider two policy scenarios specific to Rennes, both relating to city-wide goals by the year 2030. First, a policy which aims for a transportation mode share of 40% of trips by car or motorcycle, 9% of trips by cycle, 35% of trips by walking, and 16% of trips by public transit. Second, a policy which calls for the total vehicle-miles traveled (VMT) by Rennes residents to be reduced by 10% by the year 2030. We refer to these two policies as ‘Mobility’ and ‘Climate’ policies, corresponding to the mode share and VMT reduction policies, respectively.

We seek to understand the extent to which walking and cycling affect population health in Rennes. We assess the city’s current state of active travel and additionally perform scenario impact analysis of proposed policies. We use regression techniques and the HOT model with household travel survey data from Rennes to estimate public health benefits, and hope these may assist in policymaker decisions around promoting active travel in Rennes going forward.

## Methods

### Data description

We consider the 2018 household travel survey, Enquête Ménages-Déplacements, conducted by Rennes Métropole. The survey provides a single-day snapshot of individuals’ travel activity, and is carried out approximately every 10 years using a French national standard for household travel surveys ^17^. Using survey data reported from 7,978 households, we include characteristics from 10,256 participants in our health impact analyses. The following social and environmental factors are considered from the travel survey: sex, age, education, household car access, and inner-city/suburban residency.

We formulate the Rennes household travel survey for use with the Health-Oriented Transportation model. We subset this to adults aged 18–65 and differentiate between inner-city Rennes and the surrounding suburban area which is still within the metropolitan area limits. We differentiate these using the household-level ‘secteur de tirage’, which roughly translates to the inner-city vs. suburban districts. We label inner-city Rennes as “Commune de Rennes” and the surrounding suburban area as “Zone Suburbaine”. To determine weekly participation in active travel, we utilize questions specific to individuals’ walk and cycle frequency of use. Participants were asked how often they walk and cycle, for each selecting one from four options: several days per week, several days per month, occasionally, or never. We consider the first option to indicate individuals who participate weekly in active travel.

### HOT metrics

As seen in the London-based analysis by Younkin *et al*., several health-oriented transportation (HOT) metrics are introduced for estimation and communication of population-level walking and cycling analysis ^11^. “Active travel” is defined as physical activity as a result of transportation of the mode walk or cycle. Travel activity is the weekly rate of active travel in terms of Metabolic Equivalent per Task (MET)-hours per week. Prevalence of active travel is the observed one-day snapshot of the proportion of survey participants who engage in walking or cycling. This measure is built directly from the logged walk and cycle trips in the travel diary. Participation is the same proportion, but of active travelers on a weekly basis. This is estimated from participants’ responses to the multiple-choice questions of walk and cycle frequency (i.e., the proportion which selected ‘Several Days per Week’). We scale from the single-day travel diary to a weekly estimate of activity using a measure of active travel frequency. As the Rennes travel survey included a measure of weekly walk and cycle participation, we calculate frequency using the ratio between prevalence and participation. Travel intensity is defined as the mean individual travel activity achieved by a population’s active travelers, measured in MET-hours per week.

### Modeling

***Subgroup Analysis.*** We make use of three regression models, prevalence, participation, and intensity. We include social and environmental factors to control for confounding among predictors which may have a strong effect on active travel measures. We employ active travel metrics (prevalence, participation, intensity) used in the HOT statistical model ^11^. The following demographic and environmental variables are included: sex, age, education, household car access, and inner-city/suburban residency. Sex was reported as a binary variable, with participants selecting male or female. We dichotomize education and household car access. Education is split into Primary School through Secondary Baccalaureate versus Superior Baccalaureate through Apprenticeship, the latter categorized as ‘Highly Educated’. We split household car access into two categories, representing households with no personal vehicles and households with one or more personal vehicles. Survey participants who selected “NA” or did not respond to any demographic and environmental variables were not included in the analysis.

***Travel Time Substitution.*** Many government entities are prioritizing the reduction of greenhouse gas emissions to combat climate change, including investment in reductions of personal vehicle usage. We introduce the Travel Time Substitution (TTS) model, developed for quantifying the increase in active travel corresponding to a population decrease in personal motor-vehicle travel and resulting reduced vehicle miles traveled (VMT). We model a substitution of time spent in motorized travel by time spent in active travel. Let *t* = ( *t _w_*, *t _c_*, *t _o_*) be the average weekly time (in minutes) spent walking, cycling, or using other transportation modes (including all non-active travel modes). Of the non-active travel time being replaced ( *t _o_*), we assume a fraction ( *α*) of this time is replaced by an active mode, i.e., walk or cycle. The change in travel activity can then be expressed as follows: $\delta_{\mathrm{TA}}=\frac{\alpha t_{o}}{60}\left(6-3\gamma_{w}\right)$ where $\gamma_{w}=\frac{t_{w}}{t_{w}+t_{c}}$ , using 3 METs for walking and 6 METs for cycling.

***Mode Share Shift.*** As local and regional policymakers continue to promote walking and cycling for health and transport, mode share (i.e., the proportion of the population using each travel method) is increasingly used as a metric of success when evaluating a population’s levels of active travel. We introduce the Mode Share Shift (MSS) model, with which we estimate the change in travel activity associated with such a shift in walk and cycle mode share. Given the mode share at baseline *α _b_* = ( *α _w_*, *α _c_*, *α _o_*) where Σ *α _j_* = 1, we propose mode share of a proposed travel scenario different from baseline, *α _s_* = ( *α _w_* + *δ _w_*, *α _c_* + *δ _c_*, *α _o_* – *δ _w_* – *δ _c_*). Using the weekly average total number of trips, *N*, and the median duration of a trip by each mode (in minutes), *t* = ( *t _w_*, *t _c_*, *t _o_*), we estimate the associated change in population travel activity as follows: $\delta_{\mathrm{TA}}=\frac{N}{60}\left(3t_{w}\delta_{w}+6t_{c}\delta_{c}\right)$ , again using 3 METs for walking and 6 METs for cycling.

### Health estimates

***Mortality and DALY Rates.*** For estimates of 2018 mortality and disability-adjusted life year (DALY) rates in Rennes, we use the Global Burden of Disease (GBD) Results Tool, a data resource of the Global Health Data Exchange ^18^. For calculating a monetary cost corresponding to a change in travel activity and its associated health impacts, we reference the 2021 report by Daroudi *et al.* which estimates the cost per averted DALY based on various country-level metrics ^19^.

***Simplified Comparative Risk Assessment.*** Drawing on the method developed by Symonds *et al.*, we perform a simplified comparative risk assessment by computing the population attributable fraction using the estimated population mean travel activity (rather than integrating over the distribution of physical activity, as these distributions, particularly the distribution for the scenario, are not accessible) ^16^.

$$\rho =\frac{R\left(x_{b}+\delta_{\mathrm{TA}}\right)}{R\left(x_{b}\right)}-1$$

We use all-cause mortality as our response and assume the exposure-response function is exponential on a power transformed physical activity variable (MET-hours/week), *x ^p^*, i.e., *R(x)* = *e* ^– *αx ^p^*^, a similar method of linking population active transport to health impacts as with the Health Economic Assessment Tools (HEAT) and the Integrated Transport and Health Modelling Tool (ITIHIM) ^20, 21^. We fit our exponential decay curve to data from Arem *et al.* to estimate the shape parameter, *α* = 0.107, and power transformation, *p* = 0.436. The baseline physical activity for our Rennes adult population is simulated using additional data from Arem *et al.*, resulting in a baseline level of approximately *x _b_* = 12 MET-hours/week. To generate this baseline population value, we adopt both the leisure-time physical activity intervals reported by Arem *et al.* and their corresponding distribution across the authors’ overall survey population (i.e., 0.1 to <7.5, 7.5 to <15.0 MET-hours/week). We then simulate uniform distributions within each interval with *n* = 10,000 total values, ultimately calculating the median activity for our simulated distribution of individuals and their baseline physical activity ^22^.

Moving beyond estimating averted deaths as a result of active travel scenarios, we expand our methods to include measurement of DALYs. It is challenging to model the relationship between population physical activity and DALY rates. Active travel modelers have used disease-specific ERFs in order to build an estimate of DALYs, but we have not yet found a peer-reviewed methodology explicitly linking physical activity and the rate of DALYs among a large-scale, adult, urban population ^5^.

**Figure 1.**
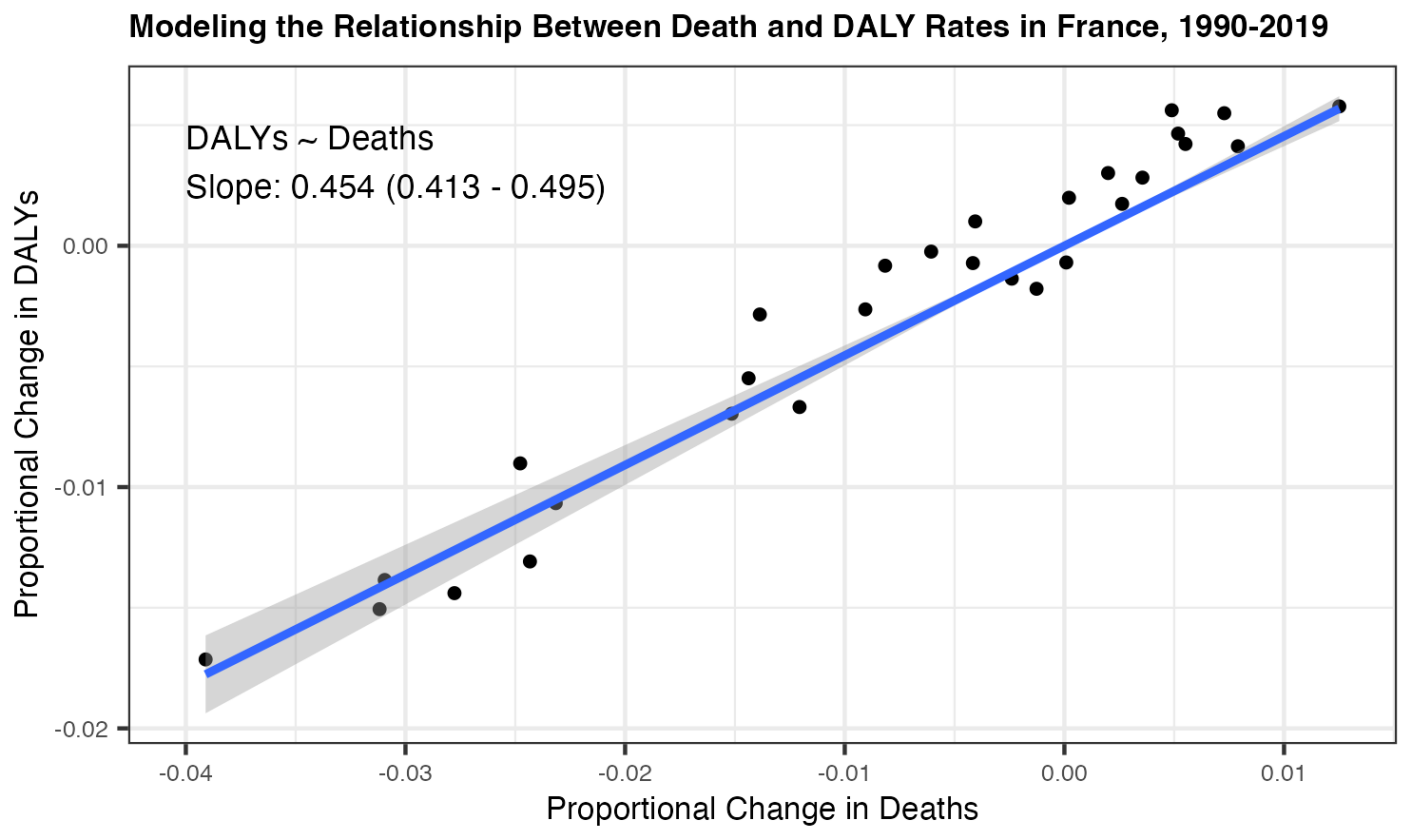
Modeling the relationship between France’s annual proportional change in deaths and DALYs for individuals aged 15–64 in years 1990–2019, Global Burden of Disease Results Tool. In addition to the visualization of death and DALY rates for those years, we include linear regression results (with intercept set to zero) demonstrating the statistically significant association between the two metrics.

To circumvent this lack of explicit measurement for an exposure-response link between population physical activity and annual DALY rates, we model the relationship between death and DALY rates in France for the years 1990–2019 (all available years in the GBD Results Tool) for individuals aged 15–64. We model the annual proportional change in deaths versus the annual proportional change in DALYs. A visualization of the death and DALY rates for those years is shown in Figure 1. A linear regression between annual proportional change in deaths and DALYs, with intercept term set to zero, results in a statistically significant linear association between the two, with the model coefficient approximately equal to 0.45 (95% CI: 0.41–0.5). As a result, for aged 15–64 individuals in France, we estimate that a 1% reduction in all-cause mortality would correspond to a 0.45% reduction in DALYs.

Building on this modeled relationship between deaths and DALYs for France, we incorporate this translation to our simplified comparative risk assessment. We utilize the Arem curve for all-cause mortality to estimate an active travel scenario’s associated impact on the Rennes population death rate by performing our simplified comparative risk assessment calculation as previously outlined. From this, we additionally compute the associated proportional change in Rennes’ rate of DALYs using our demonstrated linear association between adult death and DALY rates in France.

Increased physical activity is linked with benefiting a variety of noncommunicable health outcomes ^1^. While this translation between all-cause mortality and disability-adjusted years of life is by no means an exact measure, we acknowledge that all-cause mortality and DALYs are not built via identical algorithms, but are intended as large-scale umbrella metrics for gauging population health. Through our modeling approach we seek to translate health impacts not only to averted deaths, but averted DALYs, to better capture health impacts associated with active travel in Rennes. With a more comprehensive understanding of physical activity’s population health impacts, we show how active travel affects a more inclusive subsection of a population’s health ecosystem.

## Results

### Participant characteristics

Introductory summary statistics of participant characteristics are included in Table 1. Among 10,256 survey participants, 1,584 (15%) individuals resided in Commune de Rennes while 8,672 (85%) individuals resided in Zone Suburbaine. When considering sex, age, and education, the starkest difference was observed across age of Commune de Rennes versus Zone Suburbaine residents, with mean ages of 40 (SD = 13) and 46 (12) years, respectively. Education, in this table, includes all possible six categories, rather than its dichotomized values used in the regression analysis. When considering household car access, individual walk frequency, and cycle frequency, all three factors featured differences across inner-city and suburban survey participants. Perhaps most alarming are the reported rates of cycling frequency for ‘Several Days per Year’ and ‘Never’, totaling 76% and 84% of all individuals for both Commune de Rennes and Zone Suburban, respectively. Additionally, the lower prevalence of ‘Several Days per Week’ walk and cycle frequency among Zone Suburbaine residents (55% walk, 7.7% cycle) relative to Commune de Rennes residents (76% walk, 17% cycle) could be another possible point of focus for local health professionals.

**Table 1.** Participant Characteristics, Rennes Household Travel Survey 2018. Statistics are split between Rennes’ inner city (“Commune de Rennes”) and outer suburban area (“Zone Suburbaine”).

Participant Characteristics, EMD 2018		
Characteristic	Rennes Métropole	
	Commune de Rennes, N = 1, 584 ^1^	Zone Suburbaine, N = 8,672 ^1^
**Sex**		
M	756 (48%)	4,154 (48%)
F	828 (52%)	4,158 (52%)
**Age**	40 (13)	46 (12)
**Education**		
Primary School	18 (1.3%)	120 (1.4%)
Secondary, from 6th to 3rd	170 (12%)	1,480 (18%)
Secondary, beyond 2nd Without Completing Baccalaureate	140 (10%)	1,350 (16%)
Secondary Baccalaureate	199 (15%)	1,527 (18%)
Superior Baccalaureate, up to 2	217 (16%)	1,561 (19%)
Superior Baccalaureate, 3+ OR Apprenticeship	623 (46%)	2,327 (28%)
**Access to Car**		
Yes	607 (71%)	3,391 (93%)
No	253 (29%)	260 (7.1%)
**Walk Frequency**		
Several days per week	935 (76%)	2,990 (55%)
Several days per month	114 (93%)	674 (12%)
Several days per year	134 (11%)	1,137 (21%)
Never	46 (3.7%)	642 (12%)
**Cycle Frequency**		
Several days per week	203 (17%)	420 (7.7%)
Several days per month	101 (8.2%)	404 (7.4%)
Several days per year	216 (18%)	1,707 (31%)
Never	709 (58%)	2,912 (53%)

^1^ n (%); Mean (SD)

### Prevalence & participation

Active travel prevalence is calculated independently across all characteristics included in these analyses with weighted estimates. The overall prevalence was 24.7% (95% confidence interval: 23.7%, 25.8%). The most dramatic differences in prevalence were observed across household car access and location (inner-city vs. suburban residency). Individuals with no access to cars demonstrated an active travel prevalence of 65.3% (60.2%, 70.3%) compared to 28.6% (26.9%, 30.3%) among individuals with household access to one or more cars. Survey participants living in the inner-city, Commune de Rennes, reported a prevalence of 43.2% (40.3%, 46.2%) compared to Zone Suburbaine residents reporting 19.8% (18.7%, 20.8%).

Participation in active travel is synthesized using individuals who answered “Several Days per Week” for walking or cycling in the two survey questions and is calculated across all characteristics with weighted estimates. The overall participation was 64.5% (63.1%, 65.9%), dramatically different from the prevalence values calculated from recorded walk and cycle trips. The participation calculation excludes those who did not answer the two questions about walk and cycle frequency (54% of participants did not answer either question). This, or low frequency of weekly active travel, may account for these differences across prevalence and participation. Digging deeper into participation and differentiating between walking and cycling provides additional insight. Reported individual cycling participation, 9.7% (8.8%, 10.6%), is similar to the observed prevalence of cycling, 3.6% (3.2%, 4.1%). Examining individual weekly walk rates, however, there is a larger difference between the reported walk participation, 62.1% (60.6%, 63.5%), and the observed prevalence of walking, 46.1% (44.9%, 47.3%). If we examine the ratios between prevalence and participation (aka the HOT active travel metric ‘frequency’), these may provide more useful information. We observe the frequency for cycling to be about 0.4, whereas the same value for walking is about 0.7. This may somewhat explain the difference between observed and subjective measures of active travel in Rennes, making the raw differences less dramatic from how they may appear at first glance.

Performing a weighted chi-squared test of association for both prevalence and participation yielded statistically significant results for both household car access and inner-city residency. Additionally, education was associated with active travel prevalence. Number of survey participants in each household was added to the analysis for further insight and was not shown to be significantly associated with prevalence or participation.

### Regression analysis

Results from multivariate regression analysis are presented in Table 2, including outcomes of prevalence (multivariate logistic regression), participation (multivariate logistic regression), and intensity (multivariate log-linear regression) of active travel. The factors of household car access and inner-city residents were significant in all three regression models, while age and education (dichotomized) were additionally predictors of prevalence. For all significant factors in the three models, the resulting coefficient, whether odds ratio (prevalence, participation) or proportional change in the log-transformed coefficient (intensity), represented a positive association with active travel.

**Table 2.** Multivariate Regression Analysis of Individual Factors with Prevalence, Participation, and Intensity, Rennes Household Travel Survey 2018. Split into three regression models, highlighted rows demonstrate factors which demonstrated statistical significance. Parentheticals include 95% confidence intervals.

Multivariate Logistic Regression: Active Travel Prevalence, Participation, and Intensity Rennes Métroploe Household Travel Survey, 2018		
	Odds Ratio ^1^	p-value
Prevalence		
Female	1.07 (0.93-1.22)	0.369
Age	**1.02 (1.01-1.02)**	**< 1e-3**
Highly Educated	**1.46 (1.27-1.69)**	**< 1e-3**
No Access to Car	**4.37 (3.42-5.57)**	**< 1e-3**
Commune de Rennes Resident	**2.31 (1.93-2.75)**	**< 1e-3**
Participation		
Female	0.94 (0.83-1.06)	0.322
Age	1.01 (1-1.01)	0.105
Highly Educated	1.08 (0.95-1.23)	0.244
No Access to Car	**5.63 (4-7.92)**	**< 1e-3**
Commune de Rennes Resident	**2.73 (2.24-3.32)**	**< 1e-3**
Intensity		
Female	-0.05 (-0.16-0.08)	0.446
Age	0 (0-0.01)	0.12
Highly Educated	0.09 (-0.04-0.25)	0.173
No Access to Car	**0.56 (0.32-0.85)**	**< 1e-3**
Commune de Rennes Resident	**1.74 (1.38-2.16)**	**< 1e-3**

^1^ For Intensity, Proportional Change in Log(Intensity) is reported rather than OR.

### Policy objective: mobility

**Figure 2.**
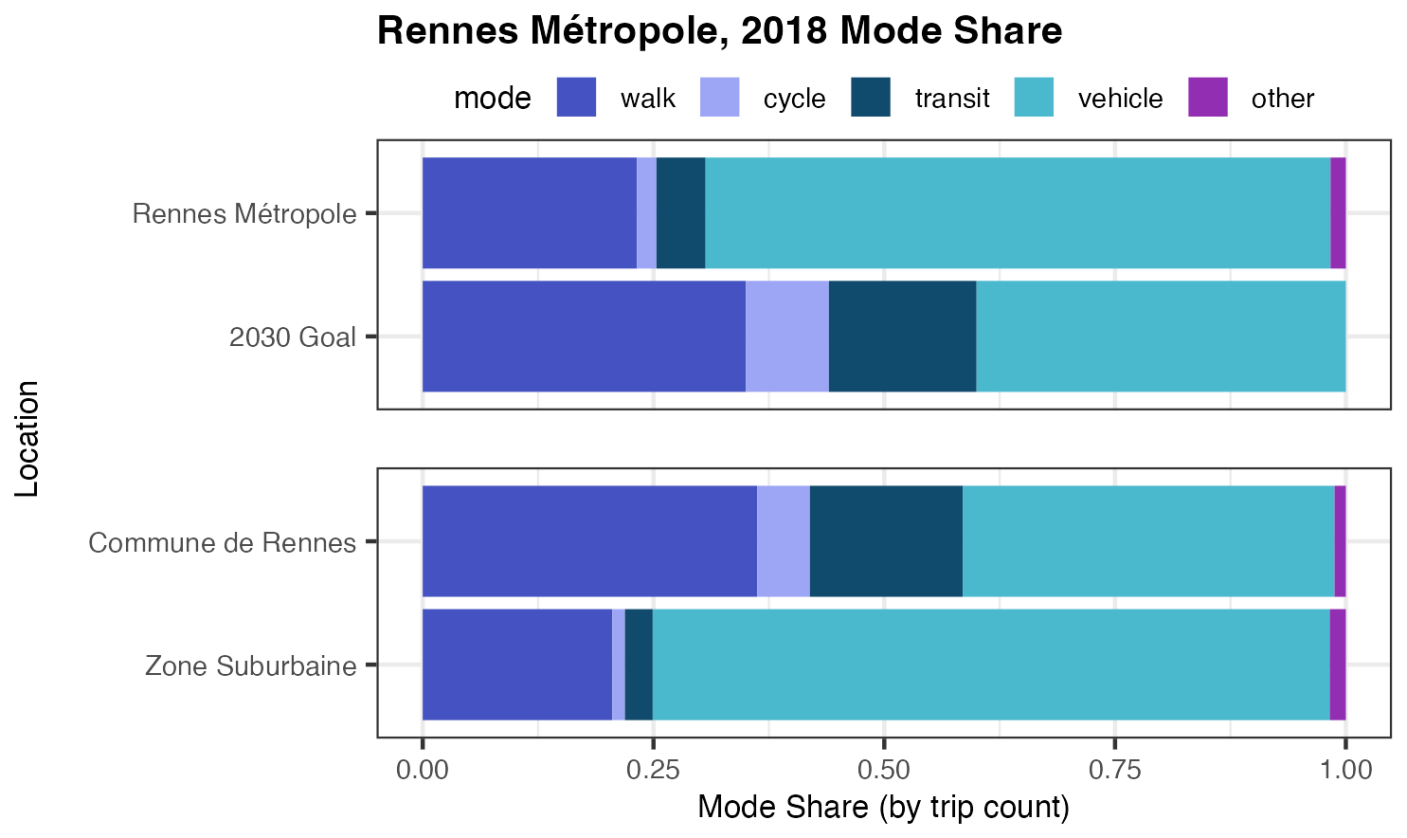
Mode Share Visualization, 2018 Rennes vs 2030 Mobility Objective, Rennes Household Travel Survey 2018. In addition to visualizing the 2018 and proposed 2030 mode shares for the entire Rennes metropolitan area, figure includes Rennes’ 2018 inner-city (“Commune de Rennes”) and suburban (“Zone Suburbaine”) comparison for greater context. The Mobility Objective’s proposed 2030 mode share aims for 40% of trips to be taken by car or motorcycle, 9% by cycle, 35% by walk, and 16% by public transit.

We consider a Rennes policy scenario designed to increase active modes of transport, specifically walking and cycling. For impact analysis of this policy, we apply the TTS and MSS models. The proposed policy would have Rennes’ mode share, by the year 2030, achieve the following: 40% of all transportation trips by car or motorcycle, 9% of trips by bicycle, 35% of trips by walk, and 16% of trips by public transit. To begin, we estimate the 2018 mode share of Rennes’ metropolitan area. This is shown in Figure 2, displaying the 2018 mode share by trip count and the 2030 mobility objective’s goal, as well as a 2018 comparison between the mode shares of subregions Commune de Rennes (City of Rennes) and Zone Suburbaine (Surrounding suburban area).

From the mode share comparison, we may observe that in order to reach the 2030 goal, the most dramatic change is required in Zone Suburbaine; in most modes Commune de Rennes is already achieving the 2030 goal. We see that Zone Suburbaine has significant ground to gain in walking, cycling, and public transit.

In Table 3, we apply the Mode Share Shift (MSS) model to the 2030 mode share scenario of 35% of trips by walk and 9% by cycle. This translates to a total of 3,319 new daily trips by walking and 1,994 trips by cycle. We estimate that the associated mean individual change in weekly activity is approximately 3.91 MET-hours/week (~40 mins of cycling/week). If Rennes were to achieve such a shift in active travel mode share usage, we estimate that this increase in activity is associated with averting 25 deaths per year or 1,051 DALYs. These averted DALYs translate to averting $73 million USD ($23–$171) in associated costs.

**Table 3.** Health and Economic Impacts of 2030 Active Travel Policies, Rennes Household Travel Survey 2018. Impacts are split into sections for each policy. The Mobility Objective section displays the impacts due to an increase in walk/cycle trips required to meet the target mode share. Within this section, the first two columns display the increase in daily walk/cycle trips associated with the Mobility Objective (proposed trip mode share of 40% car or motorcycle, 9% cycle, 35% walk, and 16% public transit). The Climate Objective requires 10% of Vehicle Miles Traveled (VMT) to be replaced by active modes. We illustrate this replacement by walk and cycle modes through various possible proportions within that replacement, each shown with their associated impacts (see first two columns). The replacement proportion of 0.89 by walk and 0.11 by cycle is representative of the current 2018 mode share in Rennes, and the 0.80/0.20 proportions correspond to the Mobility Objective’s proposed goal mode share for 2030.

Walk	Cycle	Change in Mean Travel Activity (MET-hrs/week)	Averted Deaths	Averted DALYs	Cost Reduction (USD, millions
*Mobility Objective: New Walk and Cycle Trips*					
3,319	1,994	3.91	25	1,051	$73 (23-177)
*Climate Objective: Proportion of VMT Replacement by Walking and Cycling*					
0.00	1.00	2.57	17	714	$50 (15-121)
0.80	0.20	1.55	10	442	$31 (10-75)
0.89	0.11	1.43	10	409	$28 (9-69)
1.00	0.00	1.28	9	369	$26 (8-62)

### Policy objective: climate

We consider a Rennes policy scenario in which vehicle miles traveled (VMT) are reduced by 10% by the year 2030. To estimate such a policy’s corresponding change in walking and cycling, and associated health impacts, we apply the Travel Time Substitution (TTS) model. In Table 3, we include 4 scenarios of VMT replacement: 100% cycling (an upper bound for possible new travel activity), 80% cycling and 20% walking (the 2030 mode share goal outlined in the mobility policy), 89% walking and 11% cycling (the current 2018 survey’s observed ratio of cycling to walking by trip count), and 100% walking (a lower bound for possible new travel activity). The new travel activity due to this policy scenario is shown as relative proportions of new walk and cycle trips, compared to the mobility policy objective which lists the raw total new walk and cycle trips.

For this climate objective, we estimate that the mean individual change in weekly travel activity ranges from ~1.3 MET-hours/week (30 mins of walking/week) to ~2.6 MET-hours/week (30 mins of cycling/week), corresponding to full replacement of the VMT each by walking and cycling, respectively. When 10% of Rennes’ VMT is replaced entirely by time spent walking, we estimate that this increase in activity is associated with averting 9 deaths per year or 369 DALYs. These averted DALYs translate to saving $26 million USD ($8-$62) in associated costs. When 10% of Rennes’ VMT is replaced entirely by time spent cycling, we estimate that this increase in activity is associated with averting 17 deaths per year or 714 DALYs. These averted DALYs translate to $50 million USD ($15–$121) saved. This range of measures, representing VMT replacement by some division of walking and cycling, can serve to provide lower and upper bounds for health impacts of similar transportation policies.

## Discussion

### Conclusion

With both univariate and multivariate regression analyses of Rennes subgroups, two characteristics arose as particularly influential predictors of travel activity: household car access and inner-city residency. We observed low rates of cycling among suburban survey participants. Additionally, household car access was dramatically higher in the surrounding suburban area, whose residents were also, on average, older.

Considering the 2030 policy objectives for Rennes, among suburban residents there will need to be substantive increases in active travel, particularly with cycling, in order to achieve such transformative change in both active travel modes and reduction of VMT. Achieving these goals provides a significant opportunity for not only reducing health burden on Rennes residents, but also for averting large associated costs ($104 million USD saved between both policies). Although increased active travel will require individual behavior change across populations, these improvements will not be possible without measures to support suburban active travel through innovations in infrastructure or other transportation programming.

### Limitations

The all-cause mortality dose-response curves used in the HOT statistical model are developed from large-scale cohort study data specific to adults, thus we restrict our analysis to Rennes individuals aged 18–65 ^22^. If an increase in walking and cycling is achieved among adults aged 18–65, it would not be naive to suggest that there may be some additional activity by those outside that age range who continue to engage in regular transportation trips. Additionally, the modeled relationship between death and DALY rates is based on French adults aged 15–64 in years 1990–2019. These ages do not exactly align with the 18–65 restriction of Rennes but represent the closest approximation using available data to match the same population’s demographic makeup.

We observe a difference between prevalence, built from logged walk or cycle trips in the one-day travel diary, and participation in active travel, synthesized from two individual-based survey questions about how often each individual engages in walking and cycling. Even after differentiating active travel between walking and cycling, the discrepancy between trip-based and questionnaire-based survey subsections remains. We observe much higher participation rates compared to prevalence rates and attribute this to low active travel frequency, demonstrating that a one-day trip diary does not sufficiently measure a population’s travel activity. Additionally, this difference between participation and prevalence could be due to the subjectivity of the individual-based walk/cycle participation questions, social desirability bias, or the possibility that participants may be including negligible walk trips in their assessment of how often they walk (e.g., walks from one’s apartment to the bus stop outside, or from one’s house to the household car parked outside) which were not logged in the trip diary.

The MSS and TTS models require several underlying assumptions. First, with all mode share shifts considered in these analyses, the population’s total number of transportation trips is kept constant. Second, for the climate policy objective’s VMT reduction we assume a full replacement by active modes walk and cycle, with no change in public transportation use by Rennes residents.

The ENTD data did not include many social or environmental variables such as race, ethnicity, income, or disability status, some of which have been shown to affect participation in active travel ^9^. Incorporation of these variables in the household survey could provide insight into inequities in access to active modes of travel which may contribute to health inequity.

### Public health implications

Walking and cycling mode share and distance traveled are both useful measures when considering the impact of transportation choices on population health. The city of Rennes’ goal of increasing active travel by 2030 is an important step towards improving public health. To reach their specified goals, this requires an emphasis on providing safe and easy access to active travel in the outer city. Policies targeting VMT reduction, as well as those directly aimed at changes in mode share, will not only help improve air quality and mitigate climate change, which is perhaps the primary motivation of the policies, but will also improve public health through physical activity and save money via these health impacts’ significant associated costs. Other urban policy makers would do well to adopt Rennes’ strategy to improve health through transportation. Even by simply reducing or replacing short trips in personal vehicles, there are dramatic health benefits that a population may achieve by investing in active travel ^23^.

## Data Availability

Data used in this study is licensed by a third party and is not freely available to the public. All travel diary data were de-identified prior to procurement by our research team and are licensed and maintained by the Rennes Métropole organization. Data were shared with our research team as part of our collaboration in the Complex Urban Systems for Sustainability and Health (CUSSH) Consortium alongside researchers from the École des Hautes Études en Santé Publique (EHESP), who facilitated procurement of these travel diary data for Rennes.

## Software availability

Source code available from: https://gitlab.com/GHI-UW/hot.

Archived source code at time of publication: https://doi.org/10.5281/zenodo.10436883 ^24^.
