# Supplementary material for: An Analysis of the Health Effects of Physical Activity due to Active Travel Policies in Rennes, France
Source: Wellcome Open Res. 2024 Aug 19;9:154. Originally published 2024 Mar 19. [Version 2] doi: 10.12688/wellcomeopenres.20917.2 (PMC11377920; doi:10.12688/wellcomeopenres.20917.2)
Supplement: Peer review history

# Reviewer report: Reviewer response for version 2

**DOI:** 10.21956/wellcomeopenres.25316.r111733

**Published:** 2024-12-03

**Authors:** Anna Porter

**Affiliations:** 1 Biostatistics, Ramboll U.S. Consulting, Raleigh, USA

This is a well written and comprehensive examination of the health effects of policy related proposed change in active travel in Rennes, France.

 I have a few minor suggestions: In the results section, restate what the definitions of prevalence as well as participation are.

 State the direction of the associations between sociodemographic factors (car access, inner-city residency, education) and active travel prevalence. Are these associations presented in a table? Numbers corresponding to these associations would also be useful, either in a table/figure or in the text.

 Is it stated anywhere how statistical significance is defined?

 Table 2 is confusing with OR as the heading, and intensity being proportional change. The footnote I find insufficient for addressing this. Would consider other ways to present the findings, such as presenting the three models in columns rather than rows (wide rather than long).

Is the work clearly and accurately presented and does it cite the current literature?

Yes

If applicable, is the statistical analysis and its interpretation appropriate?

Yes

Are all the source data underlying the results available to ensure full reproducibility?

Yes

Is the study design appropriate and is the work technically sound?

Yes

Are the conclusions drawn adequately supported by the results?

Yes

Are sufficient details of methods and analysis provided to allow replication by others?

Yes

Reviewer Expertise:

Physical activity epidemiology, active transportation, bicycling

I confirm that I have read this submission and believe that I have an appropriate level of expertise to confirm that it is of an acceptable scientific standard.

# Reviewer report: Reviewer response for version 2

**DOI:** 10.21956/wellcomeopenres.25316.r94013

**Published:** 2024-09-05

**Authors:** Charles Musselwhite

**Affiliations:** 1 Department of Psychology, Aberystwyth University, Aberystwyth, Wales, UK

I am satisfied with the changes made to the article which have improved the context of this important study. I visited Rennes in the summer (not for this peer review, haven't gone that far for the cause yet!). I was impressed by how easy it was to walk around the centre. No need for a car at all! But the outskirts (outer city) were pretty car dominated. I wish Rennes the best with the recommendations from this excellent study.

Is the work clearly and accurately presented and does it cite the current literature?

Partly

If applicable, is the statistical analysis and its interpretation appropriate?

Yes

Are all the source data underlying the results available to ensure full reproducibility?

Partly

Is the study design appropriate and is the work technically sound?

Yes

Are the conclusions drawn adequately supported by the results?

Yes

Are sufficient details of methods and analysis provided to allow replication by others?

Yes

Reviewer Expertise:

Environmental and Community Psychology. Transport and health. Transport and ageing. Social research methods.

I confirm that I have read this submission and believe that I have an appropriate level of expertise to confirm that it is of an acceptable scientific standard.

# Reviewer report: Reviewer response for version 1

**DOI:** 10.21956/wellcomeopenres.23143.r84551

**Published:** 2024-06-13

**Authors:** Charles Musselwhite

**Affiliations:** 1 Department of Psychology, Aberystwyth University, Aberystwyth, Wales, UK

This is a very well written paper documenting a new approach to identify potential health outcomes of transport policies and interventions. A slightly elaborated introduction to the paper would be very interesting around two points  (1) what other transport and health models are there that could have been used and how does HOT develop it and; (2) bring out the known relationships between transport and health in more detail.

 More detail on the methods would be useful: The household survey data used is 6 years out of date, so some discussion on why this is being used is needed and some discussion on what might have changed in terms of the data over the past 6 years is needed. Why only use data on 18-65 year old's? Older people being excluded from analyses is Why is US dollars rather than Euros used for health costs and benefits?

 The findings are discussed well.

 In the limitations, I understand the models have assumptions but maybe a comment on how realistic such assumptions are and how much they might influence the model outcomes would be useful (e.g. “First, with all mode share shifts considered in these analyses, the population’s total number of transportation trips is kept constant. Second, for the climate policy objective’s VMT reduction we assume a full replacement by active modes walk and cycle, with no change in public transportation use by Rennes residents.”)

 A comment on what would it take to disaggregate the outcomes by sub-groups of the population would be useful given how transport and health disadvantage follow one another.

Is the work clearly and accurately presented and does it cite the current literature?

Partly

If applicable, is the statistical analysis and its interpretation appropriate?

Yes

Are all the source data underlying the results available to ensure full reproducibility?

Partly

Is the study design appropriate and is the work technically sound?

Yes

Are the conclusions drawn adequately supported by the results?

Yes

Are sufficient details of methods and analysis provided to allow replication by others?

Yes

Reviewer Expertise:

Environmental and Community Psychology. Transport and health. Transport and ageing. Social research methods.

I confirm that I have read this submission and believe that I have an appropriate level of expertise to confirm that it is of an acceptable scientific standard, however I have significant reservations, as outlined above.

# Response

**Published:** 2024-08-14

**Authors:** Henry Fremont

**Affiliations:** University of Wisconsin-Madison, USA

We thank the reviewer for their comment about expanding the introduction to include information around existing other transport and health models, as well as more evidence surrounding the relationship between transport and health.  

 To address the comment with transport and health models, we have added the following text to the Introduction section’s third paragraph:  

*“Additional modeling approaches have been developed for estimating population active travel’s health impacts, such as the Health Economic Assessment Tool (HEAT) and the Integrated Transportation and Health Impacts Model (ITHIM). Each model includes its own set of varying assumptions and mixed methods for connecting transportation to health. Both HEAT and ITHIM incorporate air pollution and road injuries to their health outcome analysis, whereas the latest HOT statistical methods are intentionally simplified to solely include the health benefits from physical activity associated with walking and cycling.”*  

 For these two models’ citations, and for all references going forward added as a result of this revision, we note these new references in brackets alongside a corresponding citation added in the References section at the end of the document (e.g., “[NEW REFERENCE: HEAT, 2013]”). We do not include these bracketed statements in this response to reviewer document so as to avoid clutter.  

 We further include the following text at the end of the same paragraph to better illustrate how this analysis introduces novel methods compared to previous applications of HOT methods: *“Beyond our application of HOT statistical modeling to Rennes, we introduce novel methods which consider policy scenarios involving active transportation mode share and reduction in personal vehicle use.”*  

 To address the comment requesting more information surrounding the relationship between transport and health, we have added the following text to the Introduction section’s second paragraph: *“Transportation is linked to urban populations’ health through pathways of air pollution, noise, green space, injury, and physical activity.”*   This adds highly cited evidence which explicitly links transport and health through several health pathways. The Introduction section already includes numerous references which link physical (in)activity to health, as well as active travel, but did not directly link all transportation to health. Thank you for the suggestion to do so.                                                                                                            

 We appreciate the reviewer’s comment on the age of the data and to better discuss why it is being used and how the population’s travel habits may have changed since it was collected 6 years ago.  

 To address this comment, we add a new, fourth paragraph to the Limitations section: *“Analysis of Rennes’ current state of active travel might seek to utilize data more recent than 2018, but the frequency of which these EMD surveys are conducted by Rennes Métropole (historically every 10 years) led our research team to be satisfied with the 2018 travel survey’s ability to capture active travel behavior in Rennes. More recently, Buehler et al. demonstrate that in France cycling increased in 2020 compared to levels in 2019. Through research collaboration with Rennes Métropole, we are grateful for the opportunity to access these 2018 data to better understand how physical activity through travel impacts health in Rennes.”*    

 We thank the reviewer for their suggestion for the Methods section to explain our choice to subset the data to 18-65 year old participants.  We already include a paragraph addressing this at the start of the Limitations section, and do not want to repeat or move this text within the manuscript, so we have made the following change to the second paragraph of the Methods section in order to quickly summarize the justification for using 6-year-old data: *“We subset this to adults aged 18-65 and differentiate between inner-city Rennes and the surrounding area which is still within the metropolitan area limits.”*   Was changed to: *“We subset this to adults aged 18-65 to correspond to the physical activity exposure-response functions used in our health impacts analysis. We further differentiate between inner-city Rennes and the surrounding suburban area which is still within the metropolitan area limits.”*

 We are grateful for the reviewer’s question around the use of US Dollars as opposed to Euros as the currency for associated costs.   To address this comment, we add the following text to the Methods section’s *Health estimates* subsection: *“Daroudi’s report uses US Dollars (USD) as their currency to measure the cost associated with DALYs, so we continue this translation using USD as our final cost unit.”*    

 We thank the reviewer for the comment on better outlining our model assumptions and how these might influence outcomes.  To address this comment, we alter the third paragraph of the Limitations section as follows: *“The MSS and TTS models require several underlying assumptions. First, with all mode share shifts considered in these analyses, the population’s total number of transportation trips is kept constant. Second, for the climate policy objective’s VMT reduction we assume a full replacement by active modes walk and cycle, with no change in public transportation use by Rennes residents.”*   Was changed to: *“The MSS and TTS models require several underlying assumptions. First, with all mode share shifts considered in these analyses, the population’s total number of transportation trips is kept constant. As Rennes officials’ mobility policy objective outlined mode share, a proportional measure, as its outcome of choice, maintaining a constant value for total trips avoids an additional level of complexity in how the population might achieve the policy’s desired increase in walking and cycling mode share proportions. Without this constant trip total, we might consider scenarios where Rennes simply increases their number of walk or cycle trips (alongside no change in other modes), or conversely, simply decreases their non-walk and non-cycle trips (with no change in walking or cycling, meaning no change in travel activity). By keeping total transportation trips constant, we choose to avoid this ambiguity and additional dimension of analysis, through challenging this assumption provides an interesting possibility for future research. Second, for the climate policy objective’s VMT reduction we assume a full replacement by active modes walk and cycle, with no change in public transportation use by Rennes residents. We incorporate this assumption for similar reasoning as with mode share shift. Reduction in vehicle use may not directly translate to any change in active travel modes, but our analysis is concerned with just that: the health impacts of scenarios in which the VMT reduction does directly translate to an increase in walking and cycling. The additional dimensionality added by incorporating public transit is interesting but requires a level of complexity not pursued in this analysis. Future research in health-oriented transportation might consider spreading VMT reduction scenarios across walking, cycling, as well as public transit.”*   We choose to not actively investigate how “realistic” each of our scenarios might be, as we find that to be beyond the scope of our statistical modeling effort. We are translating proposed policy to estimated health impacts and believe that diving into the feasibility of these policies is certainly a very intriguing avenue for future research but is not within the bounds of this analysis.    

 We also thank the reviewer for their suggestion to discuss disaggregation of health outcomes by subgroups and how that might illuminate links between transport and health disadvantage.  To address this comment, we make the following change: *“The ENTD data did not include many social or environmental variables such as race, ethnicity, income, or disability status, some of which have been shown to affect participation in active travel. Incorporation of these variables in the household survey could provide insight into inequities in access to active modes of travel which may contribute to health inequity.”*   Was changed to: *“The EMD data did not include many social or environmental variables such as race, ethnicity, income, or disability status, some of which have been shown to affect participation in active travel. Incorporation of these variables in the household survey could allow researchers a subgroup-level measure of access to active travel, which in turn may contribute to health inequity. Active travel researchers in the future may seek to disaggregate the population health outcomes by subgroups, giving additional valuable insight into how transport affects health inequity within Rennes. This would require additional statistical tuning; while subgroups’ population totals might be usable to scale the overall health impact to specific social or environmental variables, this avenue also invites questions around the baseline distribution of physical activity of the sample population, and at this time we are not aware of any studies which measure Rennes residents’ physical activity habits at the subgroup level.”*   We correct a typo in the dataset’s acronym and suggest avenues for future research which might seek to disaggregate health outcomes by subgroups in Rennes.    

 Unrelated to any reviewer comments, we add the “EMD” acronym to the Methods section when the dataset’s name is introduced, as we reference that acronym several times later in the manuscript.     Finally, we add all relevant new citations to the bottom of the References section with suggested numbering, but we defer to editor’s citation manager for determining those. We include proper formatting for all new citations.

# Reviewer report: Reviewer response for version 1

**DOI:** 10.21956/wellcomeopenres.23143.r79273

**Published:** 2024-05-16

**Authors:** Alistair Woodward

**Affiliations:** 1 Department of Epidemiology and Biostatistics, School of Population Health, Faculty of Medical and Health Sciences, The University of Auckland, Auckland, New Zealand

A well-written and robust study. I have suggestions for minor improvements.

 Some important points and relevant additional references regarding the introduction:

 - increasing active travel reduces carbon emissions only if walking and cycling replaces travel by motor vehicles. It is easier to do the first (increase prevalence of PA), than the second (cut VMT). What is the basis for the mode share shift assumptions in Rennes?

 - it is true that few studies have looked closely at equity, but this has been done, and what's more, published work includes empirical studies which are in many ways more persuasive than modelling exercises (eg Keall et al July 2022 Transportation Research Part D)[Ref1]

 - we all hope, those of us working in the field, that documenting potential health gains of well-chosen transport policies will make it more likely that these policies are implemented. But is there any evidence that this really happens? It is 15 years since the first substantial co-benefits studies were published (eg Woodcock 2009 Lancet)[Ref 2], yet transport emissions continue upwards, Japan is, to my knowledge, the only country in which body weight has not risen, and car dominated cities prevail in must parts of the world.

 - electric bicycles out-number push bikes now, in many cities, and are the biggest selling electric vehicles world-wide. I expect similar trends apply in France? If so, the assumptions about prevalence, intensity and mode-substitution all require re-thinking. E-bikes typically require less PA per km than push bikes, but are ridden further and more frequently, and are more likely to replace vehicle trips.

 I was intrigued by the approach to estimating, indirectly, the relation between physical activity and disability adjusted life years. I presume PA will reduce DALYs only if the diseases causing disability are sensitive to the effects of exercise. Secular trends in deaths and DALYs represent the effects of all causes of ill-health, at least some of which are not relevant. (Is dementia strongly affected by physical activity, for instance?) Did the authors test the relationship for a limited range of causes of death and lost DALYs?

 Why are the health costs and gains expressed in terms of US dollars, rather than Euro? And would it be more appropriate to use metric measures such as Km rather than miles?

 In the HOT, what assumptions are made about road crash injuries associated with increased walking and cycling? And are these assumptions tuned to city circumstances? There are signs, from multi-city studies, that the changes in road trauma associated with change in active travel depend strongly on the local context, including infrastructure and vehicle fleet characteristics. (eg Stevenson 2016 Lancet)

 The relations between inner city residence, active travel and household car access are important, and expected. But what comes first? Is there any sign from the Rennes data, which are the biggest levers for transformation? Is it urban density, or car access, or popularity of walking and cycling?

Is the work clearly and accurately presented and does it cite the current literature?

Partly

If applicable, is the statistical analysis and its interpretation appropriate?

I cannot comment. A qualified statistician is required.

Are all the source data underlying the results available to ensure full reproducibility?

Yes

Is the study design appropriate and is the work technically sound?

Yes

Are the conclusions drawn adequately supported by the results?

Yes

Are sufficient details of methods and analysis provided to allow replication by others?

Yes

Reviewer Expertise:

public health, epidemiology, transport and health

I confirm that I have read this submission and believe that I have an appropriate level of expertise to confirm that it is of an acceptable scientific standard.

## References

[1] Transportation Research Part D: Transport and Environment. *https://shop.elsevier.com/journals/transportation-research-part-d-transport-and-environment/1361-9209#full-description* .2022;

[2] : Equity and other effects of a program facilitating and promoting active travel. *Transportation Research Part D: Transport and Environment* .2022;108: 10.1016/j.trd.2022.103338 10.1016/j.trd.2022.103338
